# *Snx14* Regulates Neuronal Excitability, Promotes Synaptic Transmission, and Is Imprinted in the Brain of Mice

**DOI:** 10.1371/journal.pone.0098383

**Published:** 2014-05-23

**Authors:** Hsien-Sung Huang, Bong-June Yoon, Sherian Brooks, Robert Bakal, Janet Berrios, Rylan S. Larsen, Michael L. Wallace, Ji Eun Han, Eui Hwan Chung, Mark J. Zylka, Benjamin D. Philpot

**Affiliations:** 1 Department of Cell Biology & Physiology, University of North Carolina, Chapel Hill, Chapel Hill, North Carolina, United States of America; 2 Division of Life Sciences, Korea University, Seoul, Korea; 3 Curriculum in Neurobiology, University of North Carolina, Chapel Hill, Chapel Hill, North Carolina, Unites States of America; 4 Department of Biology, University of North Carolina, Chapel Hill, Chapel Hill, North Carolina, United States of America; 5 Neuroscience Center, University of North Carolina, Chapel Hill, Chapel Hill, North Carolina, United States of America; 6 Carolina Institute for Developmental Disabilities, University of North Carolina, Chapel Hill, Chapel Hill, North Carolina, United States of America; Bilkent University, Turkey

## Abstract

Genomic imprinting describes an epigenetic process through which genes can be expressed in a parent-of-origin-specific manner. The monoallelic expression of imprinted genes renders them particularly susceptible to disease causing mutations. A large proportion of imprinted genes are expressed in the brain, but little is known about their functions. Indeed, it has proven difficult to identify cell type-specific imprinted genes due to the heterogeneity of cell types within the brain. Here we used laser capture microdissection of visual cortical neurons and found evidence that sorting nexin 14 (*Snx14*) is a neuronally imprinted gene in mice. SNX14 protein levels are high in the brain and progressively increase during neuronal development and maturation. *Snx14* knockdown reduces intrinsic excitability and severely impairs both excitatory and inhibitory synaptic transmission. These data reveal a role for monoallelic *Snx14* expression in maintaining normal neuronal excitability and synaptic transmission.

## Introduction

Both maternal and paternal autosomal chromosomes are required for embryogenesis because offspring can have imbalanced parent-of-origin-specific gene expression [1], [2]. Parent-of-origin-specific gene expression, termed genomic imprinting, results in gene products that are predominately expressed from either the maternal or paternal allele. The monoallelic expression of imprinted genes renders them particularly vulnerable to severe consequences from genetic insults. Approximately 2.5% of the genes in mice [3] and 1% of the genes in humans [4] are thought to be imprinted. A large proportion of these imprinted genes are expressed in the central nervous system where they influence brain development and function [5]. Consequently, it is not surprising that a variety of neurological disorders, including autism spectrum disorders, have been linked to misexpression of imprinted genes [6].

Despite the importance of genomic imprinting in brain function, the exact number and identity of imprinted genes continues to be debated [7]. RNA-sequencing techniques from F_1_ hybrid mice have identified many putative imprinted genes in the mouse brain [8], [9], but few of these candidates have been verified. Indeed, such verification is difficult because imprinted genes can be regulated in specific cell types and developmental stages [5]. To overcome this limitation, we modified previously employed approaches to identify neuron-specific imprinted genes [10], [11]. Using this method in mice, we identified a novel neuronally imprinted gene, sorting nexin protein 14 (*Snx14*). Sorting nexin family proteins are poorly characterized in brain. We found that SNX14 protein is increasingly expressed with brain maturation and plays a critical role in regulating neuronal excitability and synaptic transmission.

## Materials And Methods

### Mice

CAST/EiJ and BALB/c mice were purchased from the Jackson Laboratory and tissues were collected from F_1_ generation offspring between postnatal day (P) 13 to P25. Brain tissue was also collected from embryonic day (E) E15.5, young, and adult mice on a C57BL/6 strain (Charles River Laboratories). All research procedures using mice were approved by the Institutional Animal Care and Use Committee at the University of North Carolina, Chapel Hill and conformed to NIH guidelines.

### Fluorescence activated cell sorting

Neurons were separated by FACS as described [12] with minor modifications. Briefly, P13-P25 mouse brains were quickly removed and visual cortices were dissected. Visual cortices were minced on an ice-cold glass plate with scalpel blades and added to a microfuge tube with 1 ml Hibernate A medium (Hibernate-low fluorescence, Brain Bits). The medium was replaced with 1 ml of Accutase (SCR005, Millipore). Tubes were rotated for 40 min at 4°C. Tissues were pelleted by centrifugation at 450×g for 2 min and resuspended in 1 ml of Hibernate A. Cells were dissociated by multiple trituration with fire-polished glass pipettes and filtered serially through 100 µm, and then 40 µm cell strainers (BD biosciences). Filtrate was further purified by density centrifugation. The filtrate was added to the top of two layers of Percoll solutions (1.5 ml each solution) (Bottom solution: 3.600 ml Hibernate A+650.5 µl Percoll +76.5 µl 1 M NaCl, Top solution: 3.770 ml Hibernate A+480.3 µl Percoll +59.5 µl 1 M NaCl) in a 15 ml Falcon tube and centrifuged at 430×g for 3 min. The cloudy supernatant that contained mostly debris was carefully removed and the remaining solution was centrifuged at 550×g for 5 min. Cell pellets were resuspended in 0.5 ml of Hibernate A and fixed with an equal volume of ice-cold absolute ethanol on ice for 15 min. Cells were centrifuged down at 450×g for 2 min and resuspended in phosphate-buffered saline (PBS). Fixed cells were immunolabeled with anti-NeuN antibody (1∶1000, MAB377, Millipore) and subsequently with allophycocyanin-conjugated, goat anti-mouse IgG antibody (1∶1000, A-865, Invitrogen) before they were sorted using FACS Calibur (BD Biosciences) at the flow cytometry core facility, University of North Carolina.

### Real-time qPCR analysis

First-strand cDNA samples were quantified with a Nanodrop spectrophotometer (Thermo Scientific; Hudson, NH). cDNA was amplified in triplicate by quantitative PCR using SYBR Green (Sigma Aldrich; St. Louis, MO) and the ViiA 7 (Applied Biosystems; Grand Island, NY). The relative expression values were determined using *Gapdh* as a reference by the comparative Ct method (2-ΔΔCt) according to manufacturer's protocol (Applied Biosystems; Grand Island, NY).

### Imprinting analysis using restriction fragment length polymorphism (RFLP)

FACS sorted NeuN-positive and NeuN-negative cells were centrifuged at 20,000×g for 5 min at 4°C. Total RNA was extracted from cell pellets using the RNeasy Micro kit (74004, Qiagen) and reverse-transcribed into cDNA using High-Capacity cDNA Reverse Transcription kit (4368814, Applied Biosystems). A fragment of *Snx14* that contains a *Cla* I RFLP was amplified using polymerase chain reaction (PCR). The PCR product was digested with *Cla* I and resolved on 2% agarose gel. The gel was stained with ethidium bromide and the image was analyzed with imageJ.

### Fluorescence-based laser capture microdissection and sequencing

P21 mouse brains were immersed in O.C.T. compound and frozen (Tissue-Tek, SAKURA). Brains were then coronally sliced at 7 µm thickness and collected on slides (MembraneSlide 1.0 PEN Zeiss). Sections were then fixed with 100% ice-cold acetone at -20°C for two minutes and air-dried. Sections were then rinsed with DEPC and RNase inhibitor (0.5 U/µl, BioLabs, M0314S)-treated PBS, incubated with NeuN (1∶10, Millipore, MAB377) for 1 min at room temperature (RT), and washed with DEPC- and RNase inhibitor-treated PBS two times. We then incubated the sections with Alexa Fluor 546 goat anti-mouse IgG_1_(γ1) (1∶10, Invitrogen, A21123) in DEPC- and RNase inhibitor-treated PBS and washed two times with DEPC- and RNase inhibitor-treated PBS. Finally, sections were dehydrated by 75, 95, 100% ethanol and 100% Xylene and then air-dried. NeuN-positive neurons in the primary visual cortex were captured using a laser capture microscope (Zeiss PALM) and collected into AdhesiveCap 500 tubes (Zeiss). RNA was extracted by PicoPure RNA Isolation Kit (Arcturus) and amplified by RiboAmp HS Amplification Kit (MDS, Analytical Technologies). *Commd1* and *Snx14* primers used for PCR and sequencing were: *Commd1* F (5′-AAAAAGCAAGGTGGCATCAC-3′), *Commd1* R (5′-CAGTGGGCAAACAGGACTTT-3′), *Snx14* F (5′-CTAATTACGGG GTGGCTGAA-3′), *Snx14* R (5′-TGATCCTTTTGGATGGAAGC-3′).

### Primary cortical neuron cultures

E15.5 mouse cortices were dissected and seeded into poly-D-lysine (0.1 mg ml^−1^) 24-well (3×10^5^ cells/well for electrophysiology, or 1×10^5^ cells/well for staining) and 6-well (1.8×10^6^ cells/well for western blot) plates as previously reported [13].

### Western blot

Cultured neurons or brain tissues were first homogenized in lysis buffer (1% Triton X-100, 5 mM EDTA, pH 8, 0.15 M NaCl, 10 mM Tris-HCl, pH 7.5, phosphatase inhibitor cocktail 1, and protease inhibitor cocktail). To assess SNX14 protein levels, 25 µg of the total protein lysate was separated by 8% SDS-polyacrylamide gel electrophoresis. Proteins were then transferred onto nitrocellulose membranes, and immunoblotting was performed using a rabbit anti-SNX14 antibody (1∶500, Sigma, HPA017639) and Alexa Fluor 680 goat anti-rabbit IgG (Invitrogen, 1∶5,000). SNX14 protein bands were visualized using an Odyssey system (LI-COR Biosciences). We often observed two protein bands for SNX14 by Western blot, but we focused our analysis on the top band for two reasons. First, the molecular weight of the top protein band is near the predicted molecular weight (110 kDa) for SNX14. Second, samples from mouse dissociated cortical neurons consistently exhibited the top SNX14 protein band, and its levels were reduced by shRNA-mediated knockdown of *Snx14*. To control for protein loading, protein expression was expressed as a ratio of UBE3A band intensity to that of β-ACTIN (1∶5,000, Sigma, A1978), and this ratio was normalized to the group samples having the highest average intensity for that particular data set (see Figure 2A–D).

### Immunofluorescence staining

Dissociated cortical neurons were fixed with 4% paraformaldehyde in PBS for 35 min at RT, permeabilized with 0.1% Triton X-100 in PBS on ice for 20 min, and blocked with 5% goat serum with 0.1% Triton X-100 in PBS for 1 hr at RT. Neurons were then incubated with primary antibodies (SNX14, 1∶20, Sigma, HPA017639; MAP2, 1∶10,000, Abcam, ab5392) in blocking solution at 4°C for three days and then secondary antibodies (Alexa Fluor 488 Goat Anti-Rabbit H+L, 1∶250, Invitrogen, A-11008; Alexa Fluor 568 Goat Anti-Chicken, 1∶250, Invitrogen, A-11041) in blocking solution with DAPI (1∶10,000, Invitrogen, D-1306) and DRAQ5 (1∶10,000, Axxora, BOS-889-001) for 30 min at RT.

For *post hoc* staining, neurons were fixed with 4% paraformaldehyde in PBS for 35 min at RT, permeabilized with 0.3% Triton X-100 in PBS on ice for 30 min, and then incubated with Streptavidin-Alexa Fluor 568 (1∶2000, Invitrogen, S-11226) in PBS and 0.1% Triton X-100 for 30 min at RT. Neurons were then blocked in 5% goat serum and incubated with mouse anti-GAD67 (1∶500, Millipore, MAB5406) for 30 min at RT followed by Alexa Fluor 488 Donkey Anti-Mouse IgG (H+L) (1∶250, Invitrogen, A21202), DAPI (1∶10,000, Invitrogen, D-1306), and the nuclear counterstain DRAQ5 (1∶10,000, Axxora, BOS-889-001). Neurons were then washed with 0.1% Triton X-100 in PBS and mounted with Fluoro-Gel (Electron Microscopy Sciences, 17985-10). Images were acquired using a Zeiss LSM 510 confocal microscope.

### *Snx14* shRNA knockdown

Mouse lentiviral *Snx14* (Mission lentiviral transduction particle, TRCN0000316685) and non-targeted scrambled shRNAs (Mission TRC2 Control Transduction Particle puro Non-Target shRNA #1, shRNA#1) were purchased from Sigma. The MOI of *Snx14* and non-targeted scrambled shRNA are 0.75 for neurons in 6-well plates and 2.8 for neurons in 24-well plates. Mouse cortical neurons were infected with lentiviral particles at day *in vitro* (*DIV*) 4 in 250 µl (24-well plate) or 1 ml (6-well plate) of conditioned medium. Medium containing lentiviral particles was removed and replaced with conditioned medium containing 1 µg/ml puromycin at *DIV5*. Fresh medium containing 1 µg/ml puromycin was added at *DIV8* and used until further analysis at *DIV11*.

### Cell density quantification

Cortical neurons were immunostained with DRAQ5 and imaged using a Zeiss LSM 510 confocal microscope. Cells were counted automatically from images using ImageJ software.

### Voltage-clamp recordings

For miniature excitatory postsynaptic current (mEPSC) recordings, dissociated cortical neurons were placed in a submersion chamber, maintained at 30°C, and perfused at 2 ml min^−1^ with oxygenated artificial cerebrospinal fluid (ACSF) containing, in mM, 124 NaCl, 3 KCl, 1.25 NaH_2_PO_4_·H_2_O, 26 NaHCO_3_, 20 Glucose, 2 CaCl_2_, 1 MgCl_2_, and was supplemented with 200 nM tetrodotoxin (TTX, Abcam, 120055) and 50 µM picrotoxin (Sigma, P1675-5G). Neurons were visualized with an Axio Examiner (Carl Zeiss) equipped with infrared differential interference contrast optics and voltage-clamped at −70 mV. Patch pipettes were pulled from thick-walled borosilicate glass (Sutter Instrument, BF150-86-10). Open tip resistances were 2–5 MΩ when pipettes were filled with an internal solution (in mM): 100 CsCH_3_SO_3_ (Sigma, C1426), 15 CsCl (Sigma, 3309), 2.5 MgCl_2_ (Sigma, M9272), 10 HEPES (Sigma, H7523), 5 QX-314·Cl (Sigma, L1663), BAPTA-TetraCs (Invitrogen, B1212), ATP-Mg (Sigma, A9187), GTP-Na (Sigma, G8877), and 0.025 Alexa Fluor 594 (Molecular Probes, A10438). Internal solutions contained 0.05% Neurobiotin Tracer (Vector Laboratories, SP-1120) with pH adjusted to 7.25 with 1 M CsOH and osmolarity adjusted to ∼295 mOsm by addition of sucrose. Voltage-clamp recordings were performed in the whole-cell configuration using patch-clamp amplifier (Molecular Devices, MultiClamp 700B), and data were acquired and analyzed using pClamp 10.2 software (Molecular Devices). Input and series resistances were determined throughout the experiment by measuring the response to intermittent test pulses. Neurons were discarded if the series resistance was larger than 25 MΩ. Analysis was restricted to neurons that were anatomically verified by intracellular fills and biochemically confirmed to be pyramidal by *post hoc* negative staining for GAD67.

For miniature inhibitory postsynaptic currents (mIPSCs) recording, ACSF (as described above) was supplemented with 20 µM 6,7-dinitroquinoxaline-2,3-dione (DNQX) (Sigma, D0540), 100 µM D,L-2-amino-5-phosphonopentanoic acid (APV) (Abcam, 120004), and 200 nM TTX (Abcam, 120055). The same internal solution (as described above) was used and neurons were voltage-clamped at 0 mV.

Both mIPSC and mEPSC data were analyzed by an individual blinded to the experimental condition.

### Current-clamp recordings

For action potential recordings, the membrane potential was held at −70 mV and ACSF was supplemented with 20 µM DNQX, 100 µM APV, and 50 µM picrotoxin. The internal solution contained (in mM): 100 K-gluconate (Sigma, G4500), 20 KCl (Sigma, P3911), 0.2 EGTA (Fluka, 03778), 10 HEPES, Na-phosphocreatine (Sigma, P7936), 4 Mg-ATP, 0.3 Na-GTP, and 0.025 Alexa Fluor 594. Internal solutions also contained 0.05% Neurobiotin Tracer, pH was adjusted to 7.22 with 1 M KOH, and osmolarity adjusted ∼292 mOsM by addition of sucrose. Current was injected with 10 pA steps and average action potential (AP) frequency was calculated for each current injection. The spike adaptation ratio was calculated by dividing the average of the last two inter-event intervals by the first inter-event interval in the spike train. Spike trains from 10–15 Hz were averaged to calculate the spike adaptation. The voltage of spike threshold for AP generation was determined when dVm/dt reached close to 10 V/s. Resting membrane potential (V_m_) was determined before holding current was injected.

### Transfection of plasmids on dissociated cortical neurons

*DIV4* dissociated cortical neurons were used for transfection. pZsGreen1-C1-*Snx14* was made by subcloning full length *Snx14* (Open Biosystems, Clone ID: 4948538) into pZsGreen1-C1 backbone vector (Clontech, 632447). 2 µl of lipofectamine 2000 (Invitrogen, 11668-027) was added into 48 µl of Neurobasal medium (GIBCO, 12348-017) and was incubated at RT for 5 min. pZsGreen1-C1-*Snx14* plasmid (1.6 µg) was mixed with 50 µl of neurobasal medium and incubated at RT for 5 min. The diluted plasmid was combined with the diluted lipofectamine 2000 and incubated for 15 min at RT. Pre-warmed neurobasal medium (2 ml) with GlutaMax-1 (GIBCO, 35050-061) was added into the wells containing neurons. The plasmid-lipofectamine complex (500 µl) was added into the wells and incubated for 40 min. After incubation, media was removed and replaced with conditioned media. Transfected neurons were cultured for three days and then were used for further analysis.

### Statistics

Data are expressed as the mean ± s.e.m., with the sample sizes (n) shown in figures or stated in the text. Statistical analyses were performed using SigmaPlot 11 (Systat Software). Normality tests (Shapiro-Wilk) and equal variance tests were run and passed (*P*>0.05) before parametric statistical analyses were run as indicated. Otherwise, non-parametric statistical analyses were used.

## Results

### *Snx14* is a neuronally imprinted gene

We used a candidate gene approach to examine genes that were predicted to be imprinted in mice [3], and we chose to examine the imprinting status of *Snx14* because we speculated it might regulate important synaptic functions. We first examined the pattern of parent-of-origin allelic expression of *Snx14* using a polymorphic restriction site that is present in an exon of the gene. F_1_ hybrid mice from reciprocal crosses between C57BL/6 and CAST/EiJ were initially used to take advantage of the single nucleotide polymorphism between both strains; CAST/EiJ strain mice are wild-derived and have important genetic distinctions from C57BL/6 mice. To examine putative neuron-specific imprinting, we first separated cerebral cortical neurons from F_1_ hybrid mice using FACS sorting based on expression of the neuronal marker NeuN. We used quantitative PCR to demonstrate that NeuN-positive cells had little expression of the glial markers *Gfap* and *Mal* compared to NeuN-negative cells (data not shown), confirming that the FACS sorting could enrich for a largely neuronal population. We then took advantage of restriction fragment length polymorphism to show that NeuN-positive cells qualitatively exhibit a biased expression of *Snx14* from the paternal allele, and this allelic bias was not observed in NeuN-negative cells (data not shown). While this allelic bias was consistently detected over multiple trials, we failed to observe exclusive parent-of-origin-specific pattern of expression, likely due to contamination of non-neuronal cells in our PCR reaction.

**Figure 1:**
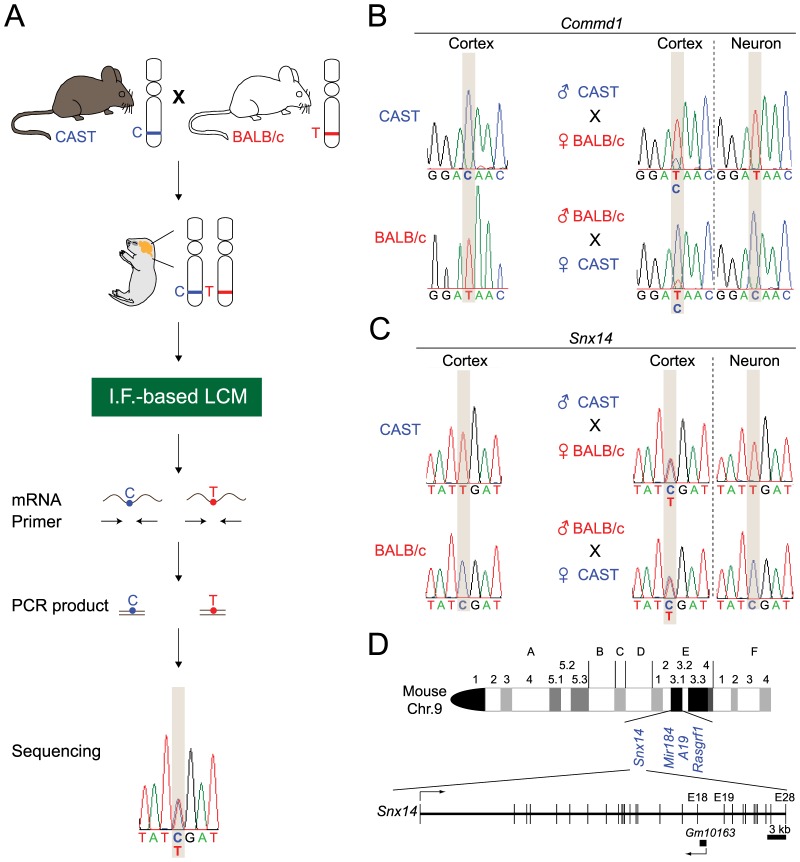
*Snx14* is imprinted in neurons. A) Method for identifying imprinted genes in neurons. Neurons were isolated from brain sections of CAST x BALB/c F_1_ offspring by immunofluorescence (I.F.)-based laser capture microdissection (LCM). Primers were designed to amplify and sequence DNA fragments containing single nucleotide polymorphisms (SNPs) within exonic regions of target genes. B) *Commd1*, a known neuron-specific imprinted gene, was used as a positive control to demonstrate that our method could detect imprinting in isolated neuron samples, but not from whole cerebral cortex homogenates containing both neurons and glia. C) SNP analyses demonstrate neuronal imprinting of *Snx14*, which is expressed from the paternal, but not maternal, allele in neurons (n = 2 mice/cross). D) *Snx14* is located within a cluster of known imprinted genes (blue). The NCBI reference sequence for *Snx14* is NM_172926.3. The NCBI reference sequence for genomic loci where *Snx14* is located is NT_039476.7.

To test for neuronal-specific imprinting of *Snx14*, it was first necessary to better isolate neurons from a heterogeneous cell population. We used immunofluorescence (I.F.)-based laser capture microdissection (LCM) to select cells labeled with the neuronal marker NeuN from the visual cortex of CAST/EiJ and BALB/c F_1_ hybrid mice. Then, mRNA was extracted from captured NeuN-positive cells (neurons) and converted into cDNA. We then used custom-made primers to amplify and sequence DNA fragments containing single nucleotide polymorphisms (SNPs) within exonic regions of target genes between these two mice strains (**Fig. 1A**). To validate this approach, we first confirmed that neurons exhibit maternal, but not paternal, allele expression of *Commd1* (**Fig. 1B**), a known neuron-specific imprinted gene. CAST and BALB/c mice carry a SNP in the exonic region of *Commd1*, such that CAST mice carry a “C” nucleotide in the region where BALB/c mice carry a “T” nucleotide. This SNP can be exploited to test whether there is parent-of-origin-specific expression of *Commd1* indicative of genomic imprinting. The maternal, but not paternal, allele expression of the SNP validated the imprinting status of *Commd1* in isolated neuron populations, but this parent-of-origin-specific expression of *Commd1* was less evident in a heterogeneous population of cells collected from the visual cortex (**Fig. 1B**). Using this approach, we observed an apparent parent-of-origin-specific expression of *Snx14* exclusively in neurons (**Fig. 1C**). Like many other imprinted genes [14], [15], *Snx14* is located near a cluster of imprinted genes of chromosome 9 (**Fig. 1D**). However, the relationship, if any, between *Snx14* and these other nearby imprinted genes such as *Rasgrf1* is currently unclear [16]. Moreover, any possible relationship between *Snx14* and *Rasgrf1* imprinting might be mouse-specific, as these genes are on different chromosomes in humans.

### SNX14 is predominantly expressed during brain development and maturation

**Figure 2:**
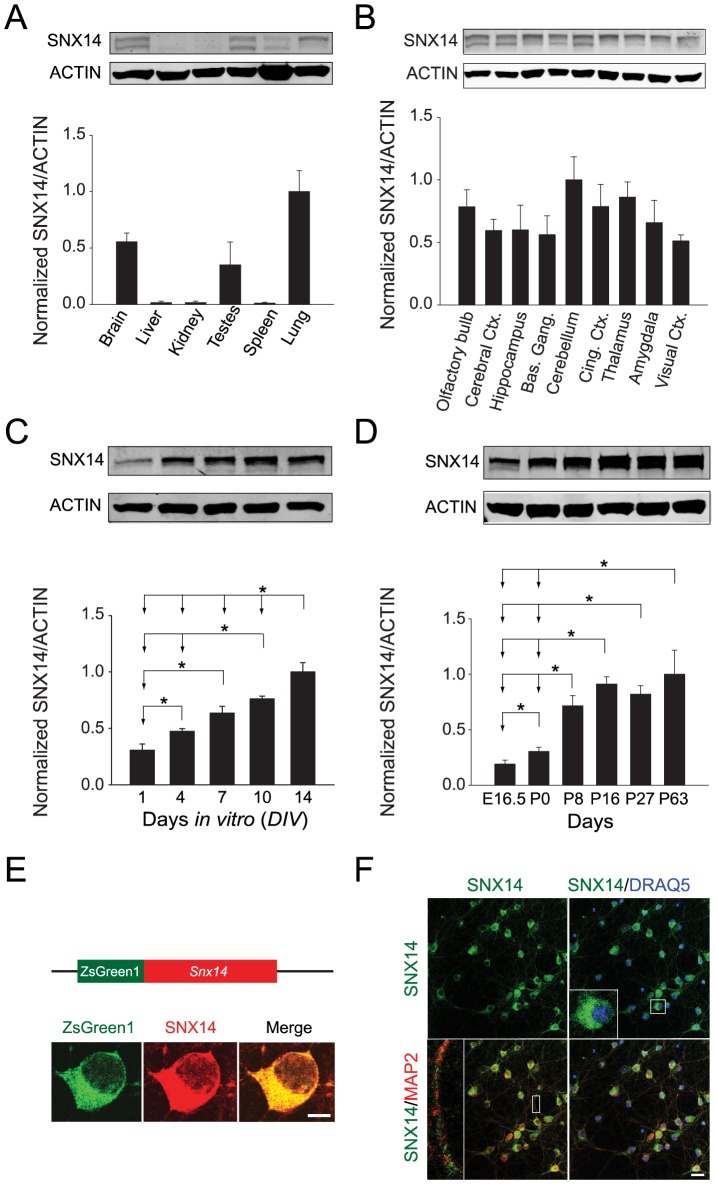
SNX14 protein levels increase during mouse brain development, and SNX14 localizes to the cytoplasm and dendrites of dissociated mouse cortical neurons. A) SNX14 protein levels were quantified by Western blotting from different mouse tissues. β-ACTIN was used as a loading control. n = 3 per group. B) SNX14 protein levels were quantified from different mouse brain regions. β-ACTIN was used as a loading control. n = 3 per brain region. Ctx.  =  cortex. Bas. Gang.  =  basal ganglia. Cing. Ctx.  =  cingulate cortex. C) SNX14 protein levels were measured in dissociated mouse cortical neurons from Days *in vitro* (*DIV*) 1 to 14. β-ACTIN was used as a loading control. *P<0.05, one-way ANOVA with Fisher LSD *post hoc*, n = 4 per age group. D) Levels of SNX14 protein were quantified in brain from embryonic to adult ages. β-ACTIN was used as a loading control. n = 6 per group. *P<0.05, one-way ANOVA with Fisher LSD *post hoc*. E) To demonstrate specificity of SNX14 antibody in cultured neurons, dissociated mouse cortical neurons were transfected with pZsGreen1-*Snx14* plasmid and subsequently immunostained with mouse anti-SNX14 antibody (red). Scale bar  = 5 µm. F) Dissociated mouse cortical neurons at *DIV* 11 were immunostained with anti-SNX14 (green), anti-MAP2 antibody (red), and counter-stained with DRAQ5 (blue). Inset on the top-right panel indicates that SNX14 is predominantly expressed in somatic cytoplasm, and the inset on the bottom-left panel shows that SNX14 is also localized to dendrites. Scale bar  = 20 µm. All bars represent mean ± s.e.m.

SNX14 protein is expressed at high levels in the brain, testes, and lung (**Fig. 2A**), and is present in diverse brain regions (**Fig. 2B**). SNX14 levels increase significantly during neuron development and maturation *in vitro* (**Fig. 2C**) and *in vivo* (**Fig. 2D**). The specificity of SNX14 antibody was confirmed by co-localization of SNX14 and its tag fusion protein, ZsGreen1 (**Fig. 2E**). In dissociated cortical neurons, immunocytochemical identification of SNX14 shows that it concentrates in the somatic cytoplasm and dendrites of dissociated cortical neurons (**Fig. 2F**). This localization and expression pattern suggests that SNX14 could play a role in brain development and maturation.

### SNX14 regulates neuronal intrinsic excitability and neurotransmission

**Figure 3:**
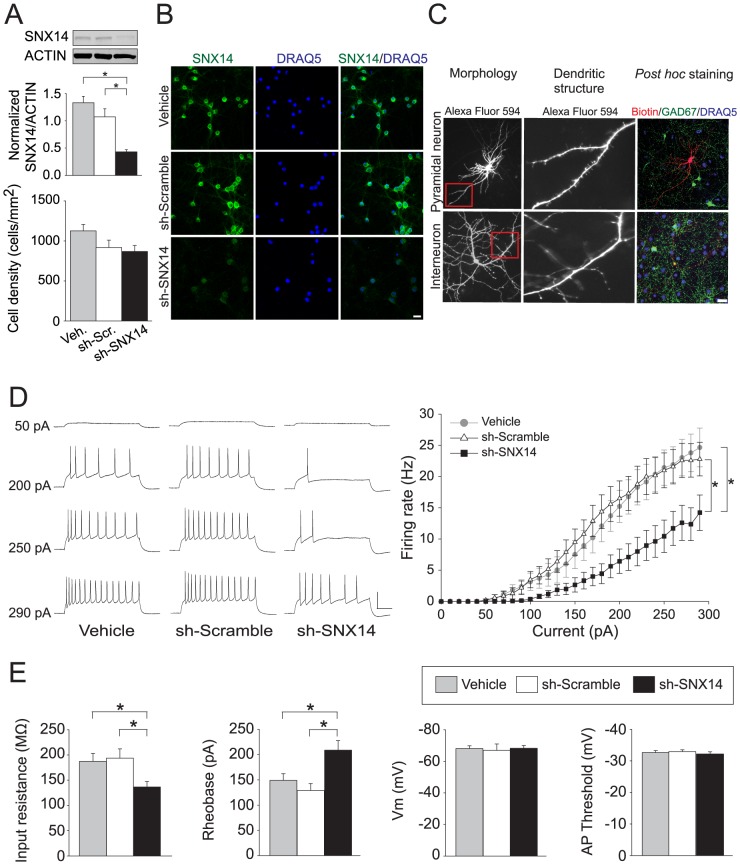
SNX14 influences intrinsic excitability of neurons. A) Dissociated mouse cortical neurons were transduced with lentiviral shRNA (scramble or *Snx14*) at *DIV* 4 and analyzed at *DIV 11* by Western blot analysis. *P<0.05, one-way ANOVA with Holm-Sidak *post hoc*, n = 4–6 per treatment. Dissociated cortical neurons were infected with lentiviral shRNA (scramble or *Snx14*) and infected neurons were selected with puromycin. No puromcyin was added into vehicle-treated dissociated cortical neurons. DRAQ5-positive cells were counted after staining. N = 20 to 25 per group. B) Dissociated mouse cortical neurons were infected with lentiviral shRNA (scramble or *Snx14*) at *DIV* 4. One week later, infected and uninfected neurons were immunostained with an anti-SNX14 antibody. Scale bar  = 20 µm. C) Dissociated mouse cortical neurons were patched with an internal solution containing Alexa Fluor 594 and biotin, and their morphology and spine structure were assessed. *Post hoc* staining for biotin and GAD67 further distinguished whether the patched cells were pyramidal neurons or interneurons. Scale bar  = 20 µm. D) Responses to current injections (left panel), and average spike frequency-current curves (right panel) from cultured pyramidal neurons treated with vehicle (n = 21), sh-Scramble (n = 20), or sh-SNX14 (n = 27) lentivirus (cells were held at −70 mV; Scale bar: 20 mv, 50 ms). *P<0.05, two-way repeated measures ANOVA with Holm-Sidak *post hoc*. E) Average input resistance, rheobase, resting membrane potential, and action potential (AP) threshold. *P<0.05, **P<0.001 one-way ANOVA with Fisher LSD *post hoc* or Kruskal-Wallis one-way ANOVA on ranks with Dunn's *post hoc*. All bars represent the mean ± s.e.m.

**Figure 4:**
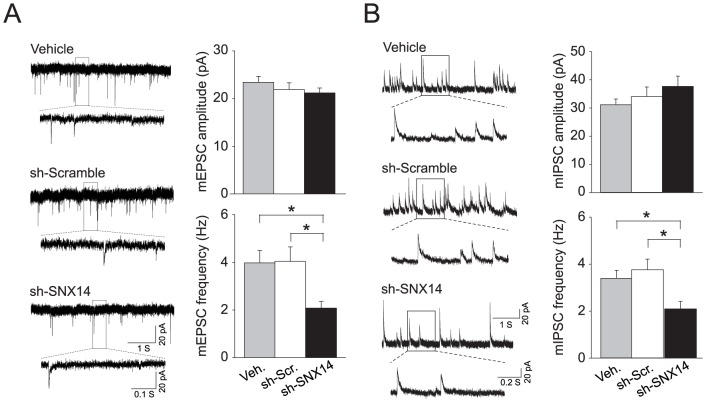
SNX14 knockdown reduces excitatory and inhibitory neurotransmission. A) mEPSC recordings demonstrating the effect of lentiviral sh-SNX14 on excitatory synaptic currents in mouse pyramidal neurons. *P<0.05, Kruskal-Wallis One Way ANOVA on Ranks with Dunn's Method *post hoc*, n = 21–25 per treatment. B) mIPSC recording demonstrating the effect of lentiviral sh-SNX14 on inhibitory synaptic currents. n = 14–18 per treatment. *P<0.05, One Way ANOVA with Holm-Sidak method *post hoc*. All bars represent the mean ± s.e.m.

Little is known about the neuronal function of sorting nexin family proteins. To determine the neuronal function of SNX14, we first verified efficient knockdown with lentiviral *Snx14* shRNAs (**Fig. 3A, B**). To limit analyses to lentiviral-infected neurons, we used *Snx14* shRNA (or scrambled control) constructs containing a puromycin resistance cassette to enable positive selection with puromycin treatment (**Fig. 3A**). We restricted our attention to pyramidal neurons, which we distinguished by their gross morphology, dendritic spines, and lack of *post hoc* staining for GAD67, a marker for inhibitory interneurons (**Fig. 3C**). Because SNX27 regulates neuronal excitability [17] and synaptic transmission [18], we speculated that SNX14 might similarly regulate neural functions. We found that *Snx14* knockdown reduced intrinsic excitability of pyramidal neurons (**Fig. 3D**) and this effect correlated with significantly decreased input resistance and increased rheobase (**Fig. 3E**). To determine if SNX14 influenced synaptic transmission, we measured miniature excitatory and inhibitory postsynaptic currents (mEPSCs and mIPSCs, respectively) from pyramidal neurons following *Snx14* knockdown. We observed a dramatic reduction in mEPSC and mIPSC frequency, but not amplitude, with *Snx14* knockdown (**Fig. 4**). These data demonstrate a significant contribution of SNX14 to intrinsic neuronal excitability and synaptic function.

## Discussion

Here we employed a screening platform to identify neuron-specific imprinted genes, and we used this technique to show for the first time the neuronal imprinting of *Snx14* in postnatal mouse visual cortical neurons. Our technique, which has been modified from previous approaches [10], [11], provides a generalizable strategy for identifying cell class-specific imprinted genes. Such studies can help bring resolution to the important debate about the number and identity of imprinted genes in the brain [7]–[9].

SNX14 belongs to the sorting nexin (SNX) protein family that includes proteins containing a phox-homology (PX) domain, which regulates adhesion to organelle membranes of the secretory and endocytic system [19]. At least 49 proteins possess a PX domain, most of them SNX protein family members, and these proteins play important roles in membrane trafficking, membrane remodeling, organelle motility, cell signaling, and protein sorting [19], [20]. Currently the contribution of SNX family members to neuronal functions or disease states is poorly understood, although there are indications that SNX family members are disrupted in patients with microcephaly [21], intellectual disability [21], and Down syndrome [18]. Misexpression of *SNX14* has itself been tentatively linked to disease. For example, dysregulation of *SNX14* has been suggested to occur in bipolar disorder [22] and 6q14 microdeletion syndrome [23]. It is interesting to speculate that the neurodevelopmental delay and intellectual disability observed in patients with a hemizygous deletion of 6q14 [23] might be caused by haploinsufficiency of *SNX14*, as there is currently no evidence that *SNX14* is imprinted in humans and we found that ∼60% knockdown of *Snx14* in mouse neurons produced dramatic phenotypic changes in neuronal intrinsic excitability and neurotransmission. While the possible linkage of SNX proteins to disease is tantalizing, there is a clear need to further elucidate the roles of SNX proteins in neurological functions and disorders.

Our developmental and spatial profiling confirmed that SNX14 is predominantly expressed in the brain, and we found that its level increased after birth and plateaued in adulthood. Given the large size of the SNX protein family, and thus the possibility for redundant functions, we were surprised that acute knockdown of *Snx14* dramatically reduced neuronal excitability and synaptic transmission. This suggests that SNX14 might have a unique and important neuronal function. Indeed, partial *Snx14* knockdown was sufficient to roughly halve the frequency of spontaneous excitatory and inhibitory synaptic events. While our findings demonstrate an important neuronal role for SNX14, future investigations are needed to distinguish the primary consequences of SNX14 loss from secondary, perhaps homeostatic, consequences. Such insights can be gained by identifying SNX14 interacting proteins, revealing the proteins trafficked by SNX14, and by understanding the temporal order of excitability and transmission deficits (e.g. does SNX14 loss first alter intrinsic excitability or synaptic transmission?). While further mechanistic insights are needed, SNX14 clearly has an important role in regulating neuronal excitability. This role for SNX14 is consistent both with its neuronal-specific imprinting and with several studies implicating other SNX proteins in regulating diverse neuronal functions such as intrinsic excitability [24] and synaptic function [17], [18], [25]. SNX might be expected to have diverse interactions, consistent with the diverse interactions of other SNX proteins such as SNX27, which regulates neuronal function through interactions with N-Methyl-d-aspartate (NMDA) receptor 2C [25] and G protein-gated inwardly rectifying potassium channels [17].

In summary, our findings suggest that *Snx14* is neuronally imprinted and critically important for normal neuronal function in mice, providing insights into neurological disorders putatively linked to dysregulation of *Snx14*. In addition, the technique employed here to identify cell type-specific imprinted genes provides a method for identifying and confirming the identity of neuronal imprinted genes.
